# Short-Term Impact of Elective Cholecystectomy on Serum Lipid Profiles: A Retrospective Cohort Study

**DOI:** 10.7759/cureus.90812

**Published:** 2025-08-23

**Authors:** Sebastien Michiels, Jean Lemaitre, Alain Van Muylem

**Affiliations:** 1 Department of Digestive and General Surgery, Clinique Sainte-Anne Saint-Rémi, Centre Hospitalier Interrégional Edith Cavell (CHIREC), Brussels, BEL; 2 Department of Digestive and General Surgery, Centres Hospitaliers Universitaires (CHU) Helora, Site Kennedy, Mons, BEL; 3 Epidemiology and Biostatistics Unit, Public Health School, Université Libre de Bruxelles, Brussels, BEL

**Keywords:** cholecystectomy, cholesterol, dyslipidemia, gallstones, lipid metabolism, lipid profile

## Abstract

Background

Gallstones are a frequent health problem and a common indication for cholecystectomy in symptomatic patients. Although gallstones are often associated with dyslipidemia, the short-term postoperative impact on lipid profiles remains unclear, with previous studies reporting conflicting results. This study evaluated the one-month effect of elective cholecystectomy on clinical and biochemical parameters, including lipid profiles.

Materials and methods

A retrospective cohort study was conducted including 61 patients who underwent elective laparoscopic cholecystectomy between October 2019 and December 2023 and had complete preoperative and one-month postoperative data. Clinical variables (age, sex, body mass index, blood pressure, heart rate) and biochemical parameters, including fasting glucose, liver enzymes, bilirubin, and lipid profiles (total cholesterol (TC), low-density lipoprotein cholesterol (LDL-c), high-density lipoprotein cholesterol (HDL-c), triglycerides (TG), Chol/HDL-c ratio), were analyzed. Paired t-tests, chi-squared tests, and logistic regression were used to assess postoperative changes and identify predictors of lipid profile improvement.

Results

No statistically significant change was observed in mean TC, TG, HDL-c, or LDL-c at one month. However, 34 patients (56%) showed a postoperative reduction in TC, while 27 (44%) did not. Higher baseline TC was the only significant predictor of improvement.

Conclusion

Elective cholecystectomy did not significantly modify lipid profiles at the group level one month postoperatively. However, patients with elevated preoperative cholesterol were more likely to experience early biochemical improvement. The predictive model developed may facilitate the identification of individuals most likely to experience metabolic benefits from the procedure. Given the short follow-up duration and limited sample size, further validation in larger prospective cohorts is warranted to confirm these findings, assess long-term outcomes, and refine predictive factors.

## Introduction

Gallstones are a prevalent global health issue, with an incidence of 1.4 per 100 person-years [1]. The condition is multifactorial, influenced by both environmental and genetic factors. Key risk factors for gallstone formation include female sex [2], increasing age [2], and dyslipidemia [2-4]. Surgical removal of the gallbladder, or cholecystectomy, is recommended for patients with symptomatic gallstones, including uncomplicated cholelithiasis and cholecystitis. Studies have shown an association between gallstones and abnormal lipid levels [5,6], which increases the risk of coronary artery disease (CAD) and stroke [7-9].

Dyslipidemia can be associated with metabolic syndrome and obesity. Some patients experience weight gain and an increase in body mass index (BMI) after cholecystectomy, which may further worsen dyslipidemic profiles [10,11].

The impact of cholecystectomy on serum lipid parameters remains unclear due to conflicting results, with some studies reporting reductions in total cholesterol (TC), triglycerides (TG), and low-density lipoprotein cholesterol (LDL-c), along with increases in high-density lipoprotein cholesterol (HDL-c) postoperatively, while others show no significant changes, particularly in the short-term period [2,4,12,13]. This variability may be partly explained by several physiological and metabolic mechanisms proposed in the literature, including alterations in bile acid circulation, lipid absorption, and dietary adaptations [4,12,14,15].

Several studies have demonstrated a statistically significant reduction in TC [12,13,16-19], TG [3,12,13,17-19], and LDL-c levels [2,3,12,13,16,17], along with an increase in HDL-c [12,13,17,18] one month post-cholecystectomy. For example, Gill and Gupta [12], Ahi et al. [13], and Al-Kataan et al. [17] examined cohorts comprising 50, 60, and 60 patients, respectively. Bistgani et al. [14] reported reductions in TC, LDL-c, HDL-c, and serum TG three days post-cholecystectomy, but no significant changes were noted at one month and one year postoperatively. Pooria et al. [4] noted a downward trend in TC one month after surgery among patients with elevated baseline cholesterol levels, though the decrease did not reach statistical significance, likely due to the small cohort size (33 patients).

More recently, a large-scale cross-sectional and Mendelian randomization analysis [20] demonstrated that cholecystectomy is causally associated with reductions in TC, LDL-c, and HDL-c, but not with glucose traits. Although our study was initiated in 2019, prior to these recent findings, our aim was to clarify the short-term metabolic consequences of cholecystectomy in a clinical cohort. While these genetic findings provide robust evidence of an association, they do not clarify the immediate postoperative metabolic effects in real-world patients.

Given the heterogeneity of previous findings, this study aims to assess short-term changes in clinical and lipid parameters and to determine whether elective cholecystectomy induces a significant improvement in the lipid profile one month after surgery in patients with symptomatic gallstone disease.

## Materials and methods

This retrospective observational study was conducted at the Department of Digestive Surgery of Iris Hospitals South (HIS) located in Brussels, Belgium. A total of 78 consecutive adult patients (aged ≥18 years) who underwent elective cholecystectomy for symptomatic gallstone disease between October 2019 and December 2023 were reviewed. All procedures were performed laparoscopically by the same experienced surgeon, ensuring consistency in operative technique and perioperative management. The study period encompassed multiple COVID-19 pandemic waves, during which surgical activity was periodically reduced.

Inclusion criteria required complete clinical and biochemical data from both the preoperative and approximately one-month postoperative periods. Patients with incomplete datasets were excluded, primarily due to missing postoperative lipid profile data (n=15). Following these exclusions, 61 patients were included in the final analysis.

The following clinical variables were collected: sex, age, body mass index (BMI), systolic and diastolic blood pressure, and heart rate. Biochemical parameters included fasting blood glucose, C-reactive protein (CRP), urea, creatinine, albumin, aspartate aminotransferase (AST), alanine aminotransferase (ALT), gamma-glutamyl transferase (GGT), alkaline phosphatase (ALP), total bilirubin, TC, LDL, HDL, cholesterol/HDL ratio, and TG. Paired t-tests were used to compare preoperative and postoperative values. For independent group comparisons, unpaired t-tests were used for continuous variables and chi-squared (χ²) tests (or Fisher's exact tests) for proportions.

Logistic regression model

Logistic regression analysis was employed to assess the impact of preoperative independent variables on the probability of improvement in lipid profiles following cholecystectomy. A univariate logistic analysis was first performed to identify variables significantly associated with the improvement in lipid profiles. Variables with a p-value of <0.15 from the univariate analysis were then included in a multivariate logistic regression model to assess their combined effect on lipid profile improvement. The final multivariate model included the following variables: CRP, creatinine, direct bilirubin, TC, the cholesterol/HDL ratio, and LDL-c. A multivariate logistic regression model was developed: $\text{Logit(improvement)}=-9.414+3.24\times\text{creatinine}+0.249\times\text{CRP}+0.032\times\text{TC}$. The probability of improvement was calculated as follows: $P=\frac{e^{\text{logit}}}{1+e^{\text{logit}}}$. The discriminative power of the logistic models was assessed using receiver operating characteristic (ROC) curve analysis. Statistical analyses were conducted using the R software (version 4.0.3, R Foundation for Statistical Computing, Vienna, Austria). A p-value of <0.05 was considered statistically significant (two-tailed).

Ethics clearance

This study was reviewed and approved by the Ethics Committee of HIS (approval number: CE/2023/10). As it involved only anonymized patient data, the requirement for informed consent was waived by the same committee in accordance with institutional policies and the Declaration of Helsinki.

## Results

A total of 78 patients were initially enrolled in the study. Seventeen were excluded, primarily due to missing postoperative lipid profile data (n=15), resulting in a final analysis including 61 patients.

Table 1 presents the preoperative characteristics of the included patients. The cohort was predominantly overweight, with a mean BMI of 28±5 kg/m² and an average body weight of 78±14 kg. Males accounted for 31.1% of the study population. Cardiovascular parameters were within expected clinical ranges, with a mean systolic blood pressure of 128±17 mmHg and a mean diastolic pressure of 78±10 mmHg. Approximately 28% of the patients were active smokers.

**Table 1:** Preoperative characteristics of the patients (n=61) Data are expressed as mean±SD for continuous variables and as number (percentage) for categorical variables. BMI: body mass index; bpm: beats per minute; WBC: white blood cell; CRP: C-reactive protein; GFR: glomerular filtration rate; AST: aspartate aminotransferase; ALT: alanine aminotransferase; ALP: alkaline phosphatase; GGT: gamma-glutamyl transferase; LDH: lactate dehydrogenase; HDL-c: high-density lipoprotein cholesterol; LDL-c: low-density lipoprotein cholesterol; Chol/HDL-c ratio: total cholesterol-to-HDL-c ratio; SD: standard deviation

Variable	Value (mean±SD or n (%))
Sex (male)	19 (31.1%)
Weight (kg)	78±14
Height (cm)	166.5±8.8
BMI (kg/m^2^)	28±5
Active smokers	17 (27.9%)
Systolic blood pressure (mmHg)	128±17
Diastolic blood pressure (mmHg)	78±10
Heart rate (bpm)	79±12
WBC count (10³/µL)	6.68±2.07
CRP (mg/L)	4.02±5.32
Albumin (g/L)	44.8±2.5
Urea (mg/dL)	31.4±15
Creatinine (mg/dL)	0.84±0.29
GFR (mL/min/1.73 m²)	91.8±24.2
AST (U/L)	22.1±19.1
ALT (U/L)	23.7±17.8
ALP (U/L)	86.4±30.7
GGT (U/L)	46±97
Total bilirubin (mg/dL)	0.47±0.22
Direct bilirubin (mg/dL)	0.21±0.08
LDH (U/L)	178.5±36.6
Total cholesterol (mg/dL)	191.6±44.2
HDL-c (mg/dL)	56.2±15.7
Chol/HDL-c ratio	3.6±1.03
LDL-c (mg/dL)	109.8±37.8
Triglycerides (mg/dL)	129±51.6
History of cholecystitis	18 (29.5%)
Glucose (mg/dL)	99.8±20.9
Diabetes	5 (8.2%)
Statin use	11 (18%)

Biological assessments indicated preserved hepatic (AST, ALT, ALP, GGT) and renal (urea, creatinine, eGFR) function, suggesting no major organ dysfunction. The inflammatory profile was only mildly disturbed, with a low mean CRP level (4.02±5.32 mg/L) and normal leukocyte counts. The lipid profile showed marked inter-individual variability, with TC ranging from 98 to 387 mg/dL, a mean HDL-c of 56.2±15.7 mg/dL, and a mean LDL-c of 109.8±37.8 mg/dL. Notably, 18% of patients were on statin therapy, and 8.2% had diabetes.

This substantial heterogeneity in preoperative TC justified a complementary analysis focused on individual postoperative changes, aiming to detect potential effects that might be masked at the group mean level.

Biochemical changes observed one month postoperatively are summarized in Table 2. Among the parameters analyzed, only ALP showed a statistically significant increase (3.3±11.2 U/L; p=0.026). Most other variables, including inflammatory, hepatic, renal, and metabolic markers, did not undergo significant changes.

**Table 2:** Mean pre- to postoperative changes at one month in patients undergoing cholecystectomy (n=61) Data are presented as mean change±SD. t-values and p-values were calculated using paired t-tests to assess the significance of changes between preoperative and one-month postoperative values. A positive mean change indicates an increase from pre- to postoperative, while a negative change indicates a decrease. CRP: C-reactive protein; GFR: glomerular filtration rate; AST: aspartate aminotransferase; ALT: alanine aminotransferase; ALP: alkaline phosphatase; GGT: gamma-glutamyl transferase; LDH: lactate dehydrogenase; HDL-c: high-density lipoprotein cholesterol; LDL-c: low-density lipoprotein cholesterol; Chol/HDL-c ratio: total cholesterol-to-HDL-c ratio; SD: standard deviation

Variable	Mean change±SD	n	t	P-value
CRP (mg/L)	0.52±8.02	61	0.51	0.618
Albumin (g/L)	-0.7±4.3	61	-1.27	0.407
Urea (mg/dL)	0±7.4	61	0.00	0.986
Creatinine (mg/dL)	1.23±9.77	61	0.98	0.329
GFR (mL/min/1.73 m²)	0.2±8	61	0.20	0.848
AST (U/L)	-2.9±18.7	61	-1.21	0.234
ALT (U/L)	-0.1±11.3	61	-0.07	0.941
ALP (U/L)	3.3±11.2	61	2.30	0.026
GGT (U/L)	-5±56	61	-0.70	0.501
Total bilirubin (mg/dL)	-0.04±0.17	61	-1.84	0.097
Direct bilirubin (mg/dL)	-0.02±0.06	61	-2.60	0.063
LDH (U/L)	-5.2±25.3	61	-1.61	0.136
Total cholesterol (mg/dL)	-6.4±31.4	61	-1.59	0.118
HDL-c (mg/dL)	-1.6±7.6	61	-1.64	0.106
Chol/HDL-c ratio	0.68±5.34	61	0.99	0.321
LDL-c (mg/dL)	-6.3±27	61	-1.82	0.078
Triglycerides (mg/dL)	14.1±56.6	61	1.95	0.056
Glucose (mg/dL)	-0.3±11.9	61	-0.20	0.870

Non-significant decreases were observed for LDL-c (-6.3±27 mg/dL; p=0.078) and HDL-c (-1.6±7.6 mg/dL; p=0.106), while TG tended to increase (14.1±56.6 mg/dL; p=0.056). Total and direct bilirubin levels slightly decreased, without reaching statistical significance. Overall, short-term changes in lipid profile and hepatic markers remained moderate, except for the increase in ALP.

To further investigate inter-individual variability, patients were stratified based on the evolution of TC following surgery. A decrease in cholesterol defined the "improvers" group (n=34), while the remainder constituted the "non-improvers" group (n=27). Pre- and postoperative changes, as well as clinical characteristics of both groups, are detailed in Tables 3-4.

**Table 3:** One-month postoperative changes in biochemical parameters among lipid profile improvers and non-improvers Data are presented as mean change±SD. Paired t-tests were used to assess changes from pre- to postoperative values within each group (t^⁎^, p^⁎^), and independent t-tests were used to compare these changes between groups (t^⁎⁎^, p^⁎⁎^). A p-value of <0.05 was considered statistically significant. CRP: C-reactive protein; GFR: glomerular filtration rate; AST: aspartate aminotransferase; ALT: alanine aminotransferase; ALP: alkaline phosphatase; GGT: gamma-glutamyl transferase; LDH: lactate dehydrogenase; HDL-c: high-density lipoprotein cholesterol; LDL-c: low-density lipoprotein cholesterol; Chol/HDL-c ratio: total cholesterol-to-HDL-c ratio; SD: standard deviation

Variable	Improvers (n=34)	t^*^	p^*^ (within-group)	Non-improvers (n=27)	t^*^	p^*^ (within-group)	t^**^	p^**^ (between-group)
CRP (mg/L)	0.44±10.33	0.25	0.808	0.62±3.84	0.83	0.412	0.08	0.933
Albumin (g/L)	-1.8±3.4	-2.01	0.056	0.9±5.3	0.56	0.585	-1.56	0.125
Urea (mg/dL)	0.3±7.2	0.21	0.837	-0.3±7.8	-0.22	0.826	0.30	0.762
Creatinine (mg/dL)	2.2±13.09	0.98	0.334	0.01±0.09	0.55	0.592	0.87	0.389
GFR (mL/min/1.73 m²)	1.4±8.1	1.05	0.304	-1.4±7.7	-0.92	0.362	1.39	0.172
AST (U/L)	-5.4±24.9	-1.24	0.223	0.1±3.4	0.23	0.820	-1.14	0.257
ALT (U/L)	-0.3±14.1	-0.13	0.895	0.2±6.3	0.14	0.891	-0.17	0.868
ALP (U/L)	3.2±11.6	1.59	0.121	3.4±11	1.64	0.114	-0.08	0.937
GGT (U/L)	-6±74	-0.48	0.634	-3±22	-0.79	0.438	-0.19	0.853
Total bilirubin (mg/dL)	-0.02±0.21	-0.54	0.595	-0.06±0.11	-2.89	0.008	0.88	0.380
Direct bilirubin (mg/dL)	0±0.06	-0.27	0.790	-0.03±0.06	-2.93	0.008	1.79	0.079
LDH (U/L)	-8.4±26.3	-1.79	0.079	-0.6±23.6	-0.12	0.903	-1.14	0.262
Total cholesterol (mg/dL)	-25.3±25.2	-5.49	<0.001	17.5±20.3	4.50	<0.001	-6.77	<0.001
HDL-c (mg/dL)	-4.7±6.5	-4.11	<0.001	2.4±7.1	1.70	0.097	-4.30	<0.001
Chol/HDL-c ratio	1.11±7.16	0.91	0.370	0.14±0.51	1.43	0.161	0.70	0.484
LDL-c (mg/dL)	-22.8±20.3	-6.29	<0.001	13.8±19.5	3.66	0.001	-6.17	<0.001
Triglycerides (mg/dL)	13.9±68.7	1.18	0.245	14.3±37.2	2.01	0.056	-0.02	0.981
Glucose (mg/dL)	0.6±12.8	0.26	0.795	-1.3±10.8	-0.61	0.551	0.59	0.556

**Table 4:** Preoperative characteristics of lipid profile improvers and non-improvers Data are presented as mean±SD for continuous variables and as number (percentage) for categorical variables. Between-group comparisons were performed using independent samples t-tests for continuous variables and chi-squared (χ²) tests for categorical variables. The specific statistical test and its corresponding test statistic (t or χ²) are provided in the "Test (statistic)" column. A p-value of <0.05 was considered statistically significant. BMI: body mass index; bpm: beats per minute; WBC: white blood cell; CRP: C-reactive protein; GFR: glomerular filtration rate; AST: aspartate aminotransferase; ALT: alanine aminotransferase; ALP: alkaline phosphatase; GGT: gamma-glutamyl transferase; LDH: lactate dehydrogenase; HDL-c: high-density lipoprotein cholesterol; LDL-c: low-density lipoprotein cholesterol; Chol/HDL-c ratio: total cholesterol-to-HDL-c ratio; SD: standard deviation

Variable	Improvers (n=34) (mean±SD or n (%))	Non-improvers (n=27) (mean±SD or n (%))	Test (statistic)	P-value
Sex (male)	10 (29.4%)	9 (33.3%)	χ²=0.00 (chi² test)	0.743
Weight (kg)	77±12	78±16	t=-0.27 (t-test)	0.868
Height (cm)	165.1±8.8	168.2±8.6	t=-1.38 (t-test)	0.179
BMI (kg/m^2^)	28±4	28±5	t=0.00 (t-test)	0.590
Active smokers	10 (29.4%)	7 (25.9%)	χ²=0.00 (chi² test)	0.763
Systolic blood pressure (mmHg)	125±12	132±22	t=-1.49 (t-test)	0.176
Diastolic blood pressure (mmHg)	78±10	80±10	t=-0.78 (t-test)	0.490
Heart rate (bpm)	79±11	77±15	t=0.58 (t-test)	0.624
WBC count (10³/µL)	6.59±2.22	6.8±1.9	t=-0.40 (t-test)	0.694
CRP (mg/L)	5.14±6.8	2.64±1.97	t=2.04 (t-test)	0.070
Albumin (g/L)	44.5±2.8	45.3±1.9	t=-1.33 (t-test)	0.349
Urea (mg/dL)	33.5±19.1	28.9±8.2	t=1.27 (t-test)	0.250
Creatinine (mg/dL)	0.9±0.36	0.77±0.13	t=1.95 (t-test)	0.078
GFR (mL/min/1.73 m²)	87.9±27.6	96.7±18.3	t=-1.49 (t-test)	0.161
AST (U/L)	23.6±25	20.2±6.7	t=0.76 (t-test)	0.503
ALT (U/L)	24.6±22.1	22.6±10.4	t=0.47 (t-test)	0.658
ALP (U/L)	83.2±27.3	90.5±34.6	t=-0.90 (t-test)	0.360
GGT (U/L)	53±127	36±33	t=0.75 (t-test)	0.505
Total bilirubin (mg/dL)	0.45±0.26	0.49±0.17	t=-0.72 (t-test)	0.490
Direct bilirubin (mg/dL)	0.19±0.07	0.23±0.09	t=-1.90 (t-test)	0.114
LDH (U/L)	176.1±34	181.4±40.3	t=-0.55 (t-test)	0.591
Total cholesterol (mg/dL)	204.4±47.7	175.4±33.6	t=2.78 (t-test)	0.010
HDL-c (mg/dL)	56.6±16.5	55.6±15	t=0.25 (t-test)	0.824
Chol/HDL-c ratio	3.81±1.01	3.33±1.01	t=1.84 (t-test)	0.073
LDL-c (mg/dL)	120.3±39.3	96.5±31.7	t=2.62 (t-test)	0.013
Triglycerides (mg/dL)	139.1±55.8	116.2±43.4	t=1.80 (t-test)	0.084
Glucose (mg/dL)	101.6±25	97.5±14.8	t=0.80 (t-test)	0.453
History of cholecystitis	8 (23.5%)	10 (37%)	χ²=0.75 (chi² test)	0.251
Diabetes	3 (8.8%)	2 (7.4%)	χ²=0.00 (chi² test)	0.841
Statin use	5 (14.7%)	6 (22.2%)	χ²=0.18 (chi² test)	0.448

Table 4 compares preoperative characteristics between the two groups. Demographic parameters, comorbidities (diabetes, smoking), blood pressure, and inflammatory markers were similar. However, baseline TC and LDL-c levels were significantly higher in the improved group compared to the non-improved group (204.4±47.7 vs. 175.4±33.6 mg/dL (p=0.010) and 120.3±39.3 vs. 96.5±31.7 mg/dL (p=0.013), respectively). This suggests that patients with a more altered initial lipid profile were more likely to derive metabolic benefit from cholecystectomy.

Other variables (CRP, creatinine, direct bilirubin, cholesterol/HDL ratio) showed trends toward between-group differences without reaching statistical significance. Statin use and the prevalence of diabetes were similar between groups, indicating that these factors did not influence lipid response.

Predictive modeling of lipid profile improvement

Logistic regression was performed to identify preoperative predictors of lipid profile improvement. Univariate analysis (Table 5) identified TC as the only significant predictive factor.

**Table 5:** Univariate and multivariate logistic regression models *: per 0.1 increase; **: per 1 unit increase; ***: per 10 unit increase

	Odds ratio (95% CI)	P-value
Univariate analysis		
Total cholesterol*** (mg/dL)	1.37 (1.14-1.77)	0.020
Multivariate analysis		
Creatinine* (mg/dL)	1.38 (1.07-1.98)	0.034
CRP** (mg/L)	1.28 (1.06-1.70)	0.030
Total cholesterol*** (mg/dL)	1.37 (1.14-1.77)	0.003

The multivariate model included variables with a p-value of <0.15 in the between-group comparisons (CRP, creatinine, direct bilirubin, TC, cholesterol/HDL ratio, and LDL-c). Table 5 presents the adjusted odds ratios (95% CI) for each included variable.

Model performance was assessed using ROC curve analysis (Table 6). The univariate model based on TC demonstrated acceptable discriminative ability, with a threshold of 195 mg/dL yielding a sensitivity of 59% and a specificity of 74%. The multivariate model showed improved performance, with a sensitivity of 85% and a specificity of 78% at a probability threshold of 0.45.

**Table 6:** Discriminant power of the logistic models AUC values with 95% confidence intervals (95% CI) for the univariate model (total cholesterol alone) and the multivariate model (total cholesterol, CRP, and creatinine). The AUC reflects the model's ability to discriminate between groups, with values closer to 1 indicating better performance. AUC: area under the curve; CRP: C-reactive protein

Model	AUC (95% CI)
Univariate model (total cholesterol)	0.69 (0.56-0.81)
Multivariate model (total cholesterol+CRP+creatinine)	0.83 (0.71-0.93)

The final logistic regression model was as follows: $\text{Logit(improvement)}=-9.414+3.24\times\text{creatinine}+0.249\times\text{CRP}+0.032\times\text{TC}$. The probability of improvement (P) was calculated using the following formula: $P=\frac{e^{\text{logit}}}{1+e^{\text{logit}}}$. These findings suggest that the proposed model could serve as a preoperative decision-support tool to identify patients most likely to experience metabolic improvement following cholecystectomy.

## Discussion

Our exploratory study sought to assess the short-term impact of cholecystectomy on lipid parameters, particularly TC, within a context of heterogeneous and sometimes contradictory findings in the literature. Several studies [4,12,14,15] have proposed that cholecystectomy may influence lipid metabolism through various mechanisms, such as accelerated enterohepatic circulation, altered bile acid synthesis, hepatic lipid remodeling, changes in fat absorption, and, potentially, dietary modifications.

These physiological adaptations could, in theory, contribute to reductions in cholesterol [12,13,16-19] or TG levels [3,12,13,17-19]. However, such effects are far from universally observed, and the evidence remains inconsistent [4,6,10,11,14,15].

More recently, a large-scale Mendelian randomization study [20] provided genetic evidence that cholecystectomy is causally associated with reductions in TC, LDL-c, and HDL-c. While these findings support a biological link between gallbladder removal and lipid metabolism, they do not clarify the short-term postoperative consequences in real-world clinical settings.

In our cohort, analysis at the group level did not reveal any statistically significant change in lipid parameters one month after cholecystectomy, suggesting that the procedure does not induce a consistent short-term lipid-lowering effect. Given the dynamic nature of lipid metabolism, the heterogeneity of individual responses, and the relatively brief one-month follow-up, this result may reflect a time frame that is too short to capture relevant metabolic changes. A delayed effect over a longer period, such as at least three months, cannot be excluded and should be investigated in future prospective studies.

To further explore the heterogeneity of responses, we performed a secondary analysis stratifying patients based on individual changes in TC levels. This post hoc classification allowed us to identify a subgroup of "improvers", patients who experienced a significant reduction in cholesterol levels after surgery. Interestingly, this subgroup tended to have higher baseline cholesterol values, a finding that aligns with previous reports [4,18,19] suggesting that baseline lipid status may modulate postoperative metabolic responses. This observation enabled us to develop a predictive model, highlighting baseline cholesterol as a potential determinant of postoperative improvement. Although statistically valid, this approach lacks established clinical relevance in the absence of external validation and long-term follow-up.

The retrospective design of our study and the selective inclusion of patients with available pre- and postoperative lipid data may introduce a substantial risk of selection bias. The term "consecutive patients" may be misleading, as only those with complete lipid datasets were analyzed. Furthermore, the sample size remains relatively small despite a long inclusion period. This limitation is partly due to the use of data from a single surgeon's practice, which reduces the cohort size but increases the reproducibility of the biological follow-up and ensures maximal inclusion of patients with complete data for statistical analyses. The low number of cases is also explained by the reduction of elective surgeries during the COVID-19 pandemic, which further affected patient recruitment.

Our logistic regression model for predicting cholesterol improvement, though statistically valid, should be considered exploratory. Without external validation and longer follow-up, its clinical applicability is limited. Additionally, no significant differences were found between improvers and non-improvers regarding cardiovascular risk factors such as BMI, hypertension, or diabetes, highlighting the complexity and multifactorial nature of lipid regulation.

Importantly, the short duration of follow-up precludes any inference about long-term cardiovascular outcomes or the sustained nature of observed biochemical changes. Dyslipidemia and its clinical implications often evolve over extended periods, and short-term fluctuations may not translate into meaningful health benefits. Therefore, based on our current data, we cannot recommend cholecystectomy as a preventive strategy for cardiovascular risk reduction.

Our cohort remains demographically representative of patients typically affected by gallstone disease, particularly with respect to age [2,4,12,14,16,17] and female predominance [2-4,12,13,16-18], as consistently reported in the literature. Although we included several relevant clinical variables associated with cardiovascular risk, further studies with larger and more diverse populations, prospective designs, and extended follow-up durations are necessary to confirm these preliminary findings and better understand the underlying biological mechanisms.

## Conclusions

In this retrospective study, elective cholecystectomy was not associated with significant short-term changes in lipid profiles at the group level. The absence of a measurable overall effect appears to conceal two distinct situations: in most patients, lipid parameters remain stable, whereas a subset, particularly those with elevated preoperative cholesterol levels, show early postoperative improvement.

These findings suggest that baseline lipid status may influence the individual metabolic response to cholecystectomy. While our logistic regression model offers a preliminary approach for identifying potential responders, its predictive value remains uncertain given the retrospective design, limited sample size, and short follow-up.

Larger prospective, longitudinal studies are needed to validate this model, clarify the underlying biological mechanisms, and determine whether early postoperative biochemical changes lead to meaningful long-term cardiovascular benefits.

At present, cholecystectomy should not be considered a therapeutic intervention for dyslipidemia or cardiovascular prevention. Nonetheless, the potential for individualized metabolic benefit supports the value of personalized assessment in the perioperative management of symptomatic gallstone disease.
